# Using Tactile Sensing to Improve the Sample Efficiency and Performance of Deep Deterministic Policy Gradients for Simulated In-Hand Manipulation Tasks

**DOI:** 10.3389/frobt.2021.538773

**Published:** 2021-06-29

**Authors:** Andrew Melnik, Luca Lach, Matthias Plappert, Timo Korthals, Robert Haschke, Helge Ritter

**Affiliations:** ^1^ CITEC, Bielefeld University, Bielefeld, Germany; ^2^ PAL Robotics, Barcelona, Spain; ^3^ OpenAI, San Francisco, CA, United States

**Keywords:** tactile sensing, robotics, reinforcement learning, shadow dexterous hand, in-hand manipulation, sample-efficiency, deep learning

## Abstract

Deep Reinforcement Learning techniques demonstrate advances in the domain of robotics. One of the limiting factors is a large number of interaction samples usually required for training in simulated and real-world environments. In this work, we demonstrate for a set of simulated dexterous in-hand object manipulation tasks that tactile information can substantially increase sample efficiency for training (by up to more than threefold). We also observe an improvement in performance (up to 46%) after adding tactile information. To examine the role of tactile-sensor parameters in these improvements, we included experiments with varied sensor-measurement accuracy (ground truth continuous values, noisy continuous values, Boolean values), and varied spatial resolution of the tactile sensors (927 sensors, 92 sensors, and 16 pooled sensor areas in the hand). To facilitate further studies and comparisons, we make these touch-sensor extensions available as a part of the OpenAI Gym Shadow-Dexterous-Hand robotics environments.

## 1 Introduction

Humans perform better in dexterous in-hand manipulation tasks than robotic systems. One of the reasons is the rich tactile perception available to humans which allows them to recognize and manipulate an object even without vision (Lederman and Klatzky, 1987). In such cases, tactile perception is one of the key abilities for in-hand manipulation of objects and tool usage. Next to vision, tactile sensing is a crucial source of information for the manipulation of objects and tool usage for humans and robots (Johansson and Westling, 1984; Maycock et al., 2010; Dang et al., 2011; Fritzsche et al., 2011). The importance of tactile sensing for object recognition was demonstrated on a multi-fingered robotic hand (Schmitz et al., 2014) as well as for successful grasping on a two-fingered gripper with high-resolution tactile sensors (Calandra et al., 2018).

**FIGURE 1:**
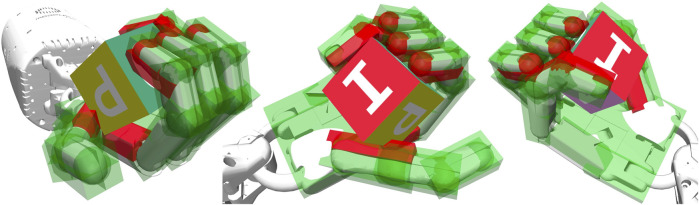
92 virtual touch sensors covering the Shadow-Dexterous-Hand model. This is a technical visualization of our sensory model. The same tactile event is shown from three different perspectives. Red sites represent activated touch sensors, where a block is pressing against the touch-sensitive area. Green sites represent inactive touch sensors. Video: https://rebrand.ly/TouchSensors.

Deep Reinforcement Learning (DRL) algorithms learn through interaction with an environment. Touch is an important sense that mediates interactions in in-hand manipulation tasks. Here we will focus on connecting DRL and tactile sensing in the context of dexterous in-hand manipulation of objects with an anthropomorphic robotic hand (Figure 1) (OpenAI et al., 2020; Plappert et al., 2018). We demonstrate that the sample efficiency and performance can be increased when tactile information is available to the agent. Different from previous works with static interaction with objects (Merzić et al., 2019), we present empirical results in a simulation that show that including tactile information in the state improves the sample efficiency and performance in dynamic in-hand manipulation tasks. To fully examine the role of tactile sensing in the improvements, we analyze the role of the spatial resolution of the tactile sensors on the hand, sensor measurement accuracy (continuous vs. Boolean values), and noise in sensory readings—a highly important aspect in using tactile sensing on physical robots. To get a better idea of useful sensor layouts and the optimal degree of sensor density, we calculated the number of activations per sensor in the 92-sensors layout (Figure 1) in the four Hand Manipulate environments: Egg, PenRotate, BlockRotateXYZ, and Block (Table 3). To assess the importance of individual tactile sensors, we also calculated the difference in Q values when dropping out an active sensor (with a Boolean sensor value). These results can guide robot engineers to build robots with tactile sensors for manipulation. We selected well-established and benchmarked simulated environments from OpenAI Gym for robotics which include in-hand manipulation of objects with anthropomorphic-hand tasks. To this end, we (i) extended the OpenAI Gym Shadow-Dexterous-Hand robotics environments with normal force touch sensors designed to simulate the touch sensing of the robot hand developed in our group (Koiva et al., 2013; Büscher et al., 2015) and (ii) compared learning results for OpenAI Gym robotics environments with and without normal force touch sensors. We find for all learning tasks a significant increase in sampling efficiency along with an improved performance of the trained agents.

An agent with a model-free policy can learn complex in-hand manipulation tasks using just proprioceptive feed-back and visual information about the manipulated object (Plappert et al., 2018). In this work, we used a combination of Deep Deterministic Policy Gradients (DDPG) (Lillicrap et al., 2016) and Hindsight Experience Replay (HER) (Andrychowicz et al., 2017). DDPG is a model-free RL algorithm for continuous action spaces employing two neural networks learning two different aspects: a target policy (also called an actor) and an action-value function approximator (called the critic). HER allows learning almost as much from achieving an undesired outcome as from the desired one and can be combined with any off-policy RL algorithm. Universal policies (Tom et al., 2015) take as input not only the current state but also a representation of the goal. The pivotal idea behind HER is to replay each episode with a different goal than the one the agent was trying to achieve, e.g. with the goals which occasionally happened to be achieved in the episode. HER is applicable when each time step has a representation in the state that can be selected as a goal. HER improves the sample efficiency and makes learning possible even if the reward signal is sparse and binary (Andrychowicz et al., 2017).

Continuous haptic feedback provides a feedback modality that can augment visual feedback, or compensate for reduced visual information under insufficient lighting or in case of visual occlusion (Korthals et al., 2019a). This can improve grasping in terms of reliability in assessing the object’s pose under conditions of uncertainty (Merzić et al., 2019). Multisensory fusion (Korthals et al., 2019b) techniques may help at the level of geometry, contact, and force physics. For example, an autoencoder can translate high-dimensional sensor representations into a lower-dimensional, compact space, easing for a reinforcement learner the task to learn stable, non-linear policies (Van Hoof et al., 2016).

**FIGURE 2:**
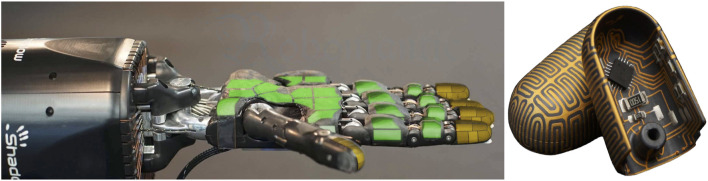
Shadow-Dexterous-Hand equipped with fabrics-based tactile sensors in the palm and finger phalanges (indicated green) and fingertip sensors realized by Molded-Interconnect-Devices (indicated yellow) (Koiva et al., 2013; Büscher et al., 2015).

Recent works describe approaches to bring tactile sensing to anthropomorphic hands like the Shadow-Dexterous-Hand, by providing integrated tactile fingertips (Koiva et al., 2013) as shown in Figure 2 and constructing a flexible tactile skin (Büscher et al., 2015). The tactile skin comprises stretchable and flexible, fabric-based tactile sensors capable of capturing typical human interaction forces within the palm and proximal and distal phalanges of the hand. This enables the hand to exploit tactile information, e.g. for contact or slip detection (Meier et al., 2016; Walck et al., 2017). Our segmentation of the simulated Shadow Dexterous Hand into 92 tactile-sensitive areas resembles the distribution of tactile sensors in these works. In Tian et al. (2019), a deep tactile model-predictive control framework for non-prehensile manipulation was proposed to perform tactile servoing and to reposition an object to user-specified configurations that were indicated by a tactile goal pattern, using the learned tactile predictive model. Van Hoof et al. (2015) studied how tactile feedback can be exploited to adapt to unknown rollable objects located on a table and demonstrated the possibility of learning feedback controllers for in-hand manipulation using reinforcement learning on an underactuated, compliant platform. The feedback controller was hand-tuned to complete the tasks.

The combination of tactile data with reinforcement learning has been explored in some previous works. In the work that investigates learning to use tactile sensing in object manipulation tasks (Chebotar et al., 2014) authors faced the problem of the high dimensionality of the tactile data. The authors showed how the learning of tactile feedback can be made more efficient by reducing the dimensionality of the tactile information through spectral clustering and principal component analysis. Another study Church et al. (2020) presented a challenging tactile robotics environment for learning to type on a braille keyboard with deep reinforcement learning algorithms. Preliminary results showed that successful learning can take place directly on a physical robot equipped with a biometric tactile sensor.

It has been shown that a model-free DRL can effectively scale up to complex manipulation tasks with a high-dimensional 24-DoF hand, and solve them from scratch in simulated experiments. With the use of a small number of human demonstrations, the sample complexity can be significantly reduced, which enables learning with sample sizes equivalent to a few hours of robot experience (Rajeswaran et al., 2017). Another study described a method for learning dexterous manipulation skills with a pneumatically actuated tendon-driven 24-DoF hand (Kumar et al., 2016). The method combined iteratively refitted time-varying linear models with trajectory optimization and can be seen as an instance of model-based reinforcement learning or as adaptive optimal control. Its appeal lies in the ability to handle challenging problems with surprisingly little data.

## 2 Methods

### 2.1 Simulator

OpenAI Gym (Brockman et al., 2016) contains several simulated robotics environments with the Shadow-Dexterous-Hand model. Simulation is carried out using the MuJoCo physics engine (Todorov et al., 2012). These are open-source environments designed for experimental work and research with Deep Reinforcement Learning. The anthropomorphic Shadow-Dexterous-Hand model, comprising 24 degrees of freedom (20 actuated and four coupled), has to manipulate an object (block, egg, or pen) so that it matches a given goal orientation, position, or both position and orientation. For the original environments without touch sensing, the state vector is 68-dimensional (Table 1) (Plappert et al., 2018). The state vector includes the 24 positions and 24 velocities of the robot’s joints. It also includes the Cartesian position and rotation of the object that is being manipulated, represented by a unit quaternion as well as its linear and angular velocities.

**TABLE 1:** Neural Network Input Vector in the Hand Environments.

Type	92 sensors	16 sensors
Joint angles	24	
Joint angles’ velocity	24	
Object’s position XYZ	3	
Object’s velocity XYZ	3	
Object’s orientation (quaternion)	4	
Object’s angular velocities	3	
Target position of the object XYZ	3	
Target orientation of the obj. XYZ (quaternion)	4	
Vector length without touch sensors	68	
Vector length with touch sensors	160	84

### 2.2 Touch Simulation

We covered all five fingers and the palm of the Shadow-Dexterous-Hand model with 92 (Figure 1; Table 2) and 927 virtual touch sensors. For the agent, the only difference between the robotics environments with and without touch sensors is the length of the state vector that the agent receives as input at each time step (Table 1). In the environments extended with 92 touch sensors, the state vector is 160-dimensional (68 + 92) (Table 1). In the environments extended with 927 touch sensors, the state vector is 995-dimensional (68 + 927).

**TABLE 2:** The 92 and 16 Touch-Sensor Environments.

	Sensors-per-area × number-of-areas	
Functional areas of the hand model	92-Sens. Model	16-Sens. Model
Lower phalanx of the fingers (× 4)	7 sensors × 4	1 sensor × 4
Middle phal. of the fingers (× 4)	5 sensors × 4	1 sensor × 4
Tip phalanxes of the fingers (× 4)	5 sensors × 4	1 sensor × 4
Thumb phalanxes (× 3)	5 sensors × 3	1 sensor × 3
Palm (× 1)	9 sensors × 1	1 sensor × 1

As an additional experiment, we grouped 92 sensors into 16 sub-groups (Table 2) to reduce the tactile sensory resolution when using Boolean tactile signals. In the environments with 16 touch-sensor sub-groups the state vector is 84-dimensional (68 + 16) (Table 1). If any of the sensors in a group has a greater than zero value, then the tactile sub-group returns 1, otherwise 0. The grouping was done per phalanx (3 phalanxes × 5 digits) plus a palm resulting in 16 sub-groups (Table 2).

**FIGURE 3:**
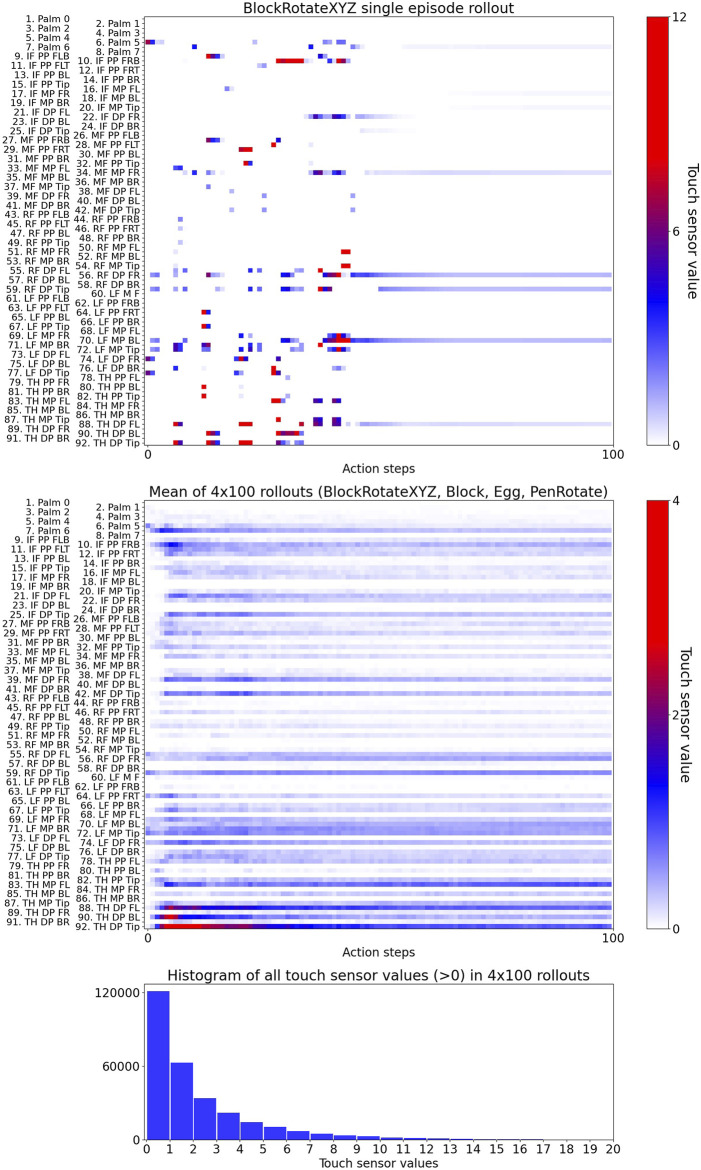
Visualisation of touch-sensor values in a single episode rollout (upper plot), mean of 400 rollouts (middle plot), and a histogram of all touch-sensor values in the 400 rollouts. IF - Index Finger, MF - Middle Finger, RF - Ring Finger, LF - Little Finger, DP - Distal Phalanx, MP - Middle Phalanx, PP - Proximal Phalanx, FL - Front Left, FLB - Front Left Bottom, FLT - Front Left Top, FR - Front Right, FRB - Front Right Bottom, FRT - Front Right Top, BL - Back Left, BR - Back Right.

The MuJoCo (Todorov et al., 2012) physics engine provides methods to mimic touch sensing at specified locations. This is based on specifying the tactile sensors’ active zones by so-called sites. Each site can be represented as either ellipsoid or box. In Figure 1, the sites are visualized as red and green transparent shapes attached to the hand model. If a body’s contact point falls within a site’s volume and involves a geometry attached to the same body as the site, the corresponding contact force is included in the sensor reading. Soft contacts do not influence the above computation except inasmuch as the contact point might move outside of the site, in which case if a contact point falls outside the sensor zone, but the normal ray intersects the sensor zone, it is also included. MuJoCo touch sensors only report normal forces using Minkowski Portal Refinement approach (Gary, 2008a; Gary, 2008b; Michael and Newth, 2013), not the Separating Axis Theorem, and friction does not play a role. The output of the contact sensor is a non-negative scalar value of type float that is computed as the sum of all contact normal forces that were included for this sensor in the current time step (Todorov, 2019a; Todorov, 2019b). Thus, each sensor of the 92 virtual touch sensors has a non-negative scalar value (Figure 3). In experiments E3 and E4, we applied a threshold and used Boolean value for each sensor, i.e., in-contact and not-in-contact.

### 2.3 Reinforcement Learning and Training

We evaluate learning using Deep Deterministic Policy Gradients (DDPG) (Lillicrap et al., 2016) and Hindsight Experience Replay (HER) (Andrychowicz et al., 2017) techniques. For a given state of the environment, a trained policy outputs an action vector of 20 continuous values ranging from -1 to 1 used for position-control (actuation center + action × actuation range/2) for 20 actuated degrees of freedom (non-coupled joints). The learning agent in the environment is an Actor and Critic network. All experiments in this paper use the following hyperparameters:

• Actor and critic networks: 3 layers with 256 units each and ReLU non-linearities
• Adam optimizer (Kingma and Jimmy, 2014) with $10^{-3}$ for training both actor and critic
• Buffer size: $10^{6}$ transitions
• Polyak-averaging coefficient: 0.95
• Action L2 norm coefficient: 1.0
• Observation clipping: [−200, 200]
• Batch size: 256
• Rollouts per MPI worker: 2
• Number of MPI workers: 19
• Cycles per epoch: 50
• Batches per cycle: 40
• Test rollouts per epoch: 10
• Probability of random actions: 0.3
• Scale of additive Gaussian noise: 0.2
• Probability of HER experience replay: 0.8
• Normalized clipping: [−5, 5]

These hyperparameter values and the training procedure are the same as in Plappert et al. (2018) and described there in greater detail, also available as a part of the OpenAI Baselines^1^. However, experiments in Plappert et al. (2018) were conducted without tactile information.

Following Plappert et al. (2018), in all tasks, rewards are sparse and binary: At each timestep, the agent receives a reward of 0 if the goal has been achieved (within some task-specific tolerance), and −1 otherwise. An episode is considered successful if, at the last time step of the episode, the manipulated object is in the goal state (within some task-specific tolerance).

For all environments, we train on a single machine with 19 CPU cores, one worker per CPU core. Workers use Message Passing Interface (MPI) for synchronization. Each MPI worker generates two rollouts of experience per cycle. MuJoCo parallel simulation environments were the main time-limiting factor and not the DRL algorithm computation, therefore the experiments were done on a CPU cluster. One epoch consists of 2 (rollouts per worker) × 50 (cycles) × 19 (CPUs) = 1,900 full episodes. One episode consists of 100 timesteps, thus, one epoch contains 190,000 samples. In “HandManipulateEgg” and “HandManipulatePenRotate” environments we train for 1,000 epochs, which amounts to a total of 19 × 10^7^ timesteps. In the “HandManipulateBlockRotateXYZ” environment we trained the agent for 300 epochs, which amounts to a total of 5.7 × 10^7^ timesteps. In the “HandManipulateBlock” environment we trained the agent for 2000 epochs, which amounts to a total of 38 × 10^7^ timesteps. All environments ran at 500 frames per second (FPS). We apply the same action in 20 subsequent simulator steps (frameskip = 20), with Δt = 0.002 s each, before returning control to the agent, i.e., the agent’s action frequency is f = 25 Hz (25 timesteps per second). In all cases, we repeat an experiment with 5 different random seeds and report results by computing the median test success rate as well as the interquartile range. We evaluate the performance after each epoch by performing 10 deterministic test rollouts per MPI worker and then compute the test success rate by averaging across rollouts and MPI workers.

### 2.4 Task Environments

As a step toward touch-augmented RL, we extended the Shadow-Dexterous-Hand model with touch sensors and made it available in new “TouchSensors” environments (Table 3) in the OpenAI Gym (Brockman et al., 2016)^2^. “…TouchSensors-v0” environments contain a vector of 92 Boolean values representing tactile information. If a sensor has a greater than zero value, then for this sensor the environment returns 1, otherwise 0. “…TouchSensors-v1” environments contain a vector of 92 continuous values representing tactile information.

**TABLE 3:** New OpenAI Gym Robotics Environments with 92 Touch Sensors: –v0 (Boolean), –v1 (Continuous-Value).

Environment name
HandManipulate**BlockRotateZ**TouchSensors
HandManipulate**BlockRotateParallel**TouchSensors
HandManipulate**BlockRotateXYZ**TouchSensors
HandManipulate**Block**TouchSensors
HandManipulate**EggRotate**TouchSensors
HandManipulate**Egg**TouchSensors
HandManipulate**PenRotate**TouchSensors
HandManipulate**Pen**TouchSensors

**FIGURE 4:**
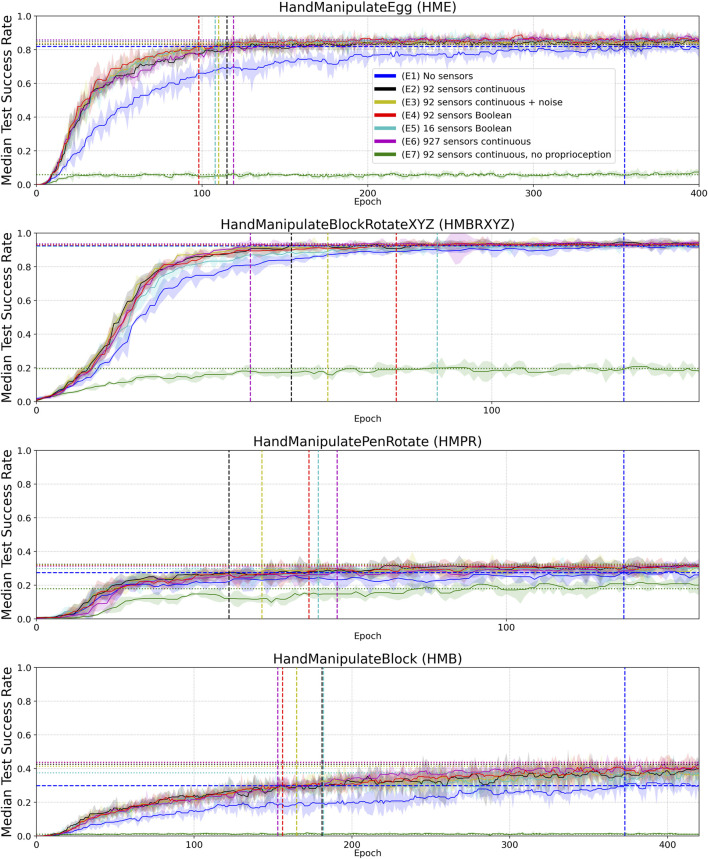
Median Success Rate curves (vertical axes) vs. training epochs (horizontal axes) for tasks HME, HMBRXYZ, HMPR, HMB and experiments E1–E7 (colored curves–see HME figure legend). See Figure A1 in Appendix for the full convergence tails. Each curve shows the median success rate across five different training trials with a different random seed. The success rate value in each training trial is the mean of 10 tests after each training epoch. The median smoothing window of plotted curves is equal to five epochs. Shaded areas represent the interquartile range. Horizontal dotted lines highlight the success-rate-convergence level of corresponding (color) curves (Table 4). The blue horizontal dashed line highlights the success-rate-convergence level of learning without touch sensors (No sensors) (Table 5). Vertical dashed lines (blue, black, yellow, red, and green) highlight the intersection of corresponding curves (color) with the blue dashed line (Table 5).

For evaluation of sample efficiency and performance with and without tactile information, we selected four environments: ”HandManipulateEgg”, “HandManipulatePenRotate”, “HandManipulateBlock”, and “HandManipulateBlockRotateXYZ”. On the one hand, we intended to cover a wide range of environments with different learning complexities and various objects for manipulation. On the other hand, we had limited computational resources, and therefore we could not conduct experiments for all possible “TouchSensors” environments and combinations of various factors and parameters. The main limiting factor is to run the MuJoCo simulator, which runs on the CPU. For one machine with 20 CPU cores, it takes more than one year of compute time for experiments (E1-E10) in the paper. In Figure 4: approximately 358 days = 3 min per epoch × 5 seeds × 7 experiments × (300 + 1,000 + 1,000 + 2,000) epochs in four environments. In Figure 6: approximately 134 days = 3 min per epoch × 5 seeds × 3 experiments × (300 + 1,000 + 1,000 + 2,000) epochs in four environments.

#### 2.4.1 HandManipulateEgg (HME)

The task is to manipulate the egg so that the egg reaches its target pose. The goal is represented as a 7-dimensional vector and includes the target position (3 Cartesian coordinates) and target rotation (4 quaternion values) of an egg-shaped object. Thus, the goal has six independent degrees of freedom, as one of the quaternion components is not independent. The rotational symmetry of the egg does not count in this case, as the surface of the egg is marked with letters. At the beginning of each episode, a random target rotation for all axes of the object and a random target position are selected. The goal is considered achieved if the distance between the manipulated objects position and its desired position is less than 1 cm and the difference in the rotation is less than 0.1 rad.

#### 2.4.2 HandManipulatePenRotate (HMPR)

The task is to manipulate the pen so that the pen reaches its target pose. The goal is represented as a 4-dimensional vector and includes the target rotation (4 quaternion values) of a pen-shaped object. The pen has rotational symmetry. Thus, the goal has 2 independent degrees of freedom, as two of the quaternion components are not independent. At the beginning of each episode, a random target rotation of the object for *x*- and *y*- axes and no target rotation around the *z*-axis is selected. No target position is selected. The goal is considered achieved if the difference in rotation, ignoring the *z*-axis, is less than 0.1 rad.

#### 2.4.3 HandManipulateBlock (HMB)

The task is to manipulate the block so that the block reaches its target pose. The goal is represented as a 7-dimensional vector and includes the target position (3 Cartesian coordinates) and target rotation (4 quaternion values) of a block-shaped object. Thus, the goal has six independent degrees of freedom, as one of the quaternion components is not independent. At the beginning of each episode, a random target rotation for all axes of the object and a random target position are selected. The goal is considered achieved if the distance between the manipulated objects position and its desired position is less than 1 cm and the difference in the rotation is less than 0.1 rad.

#### 2.4.4 HandManipulateBlockRotateXYZ (HMBRXYZ)

The task is to manipulate the block so that the block reaches its target rotation. The goal is represented as a 4-dimensional vector (4 quaternion values) of the target rotation of a block-shaped object. Thus, the goal has 3 independent degrees of freedom, as one of the quaternion components is not independent. At the beginning of each episode, a random target rotation for all axes of the object and no target position are selected. The goal is considered achieved if the difference in the rotation is less than 0.1 rad.

For the sake of brevity, further details about training procedure, reward function, goal-aware observation space, and neural network parameters are available in Plappert et al. (2018). Our main contribution focuses on the extension of the existing Shadow-Dexterous-Hand model by tactile sensors and providing insights about how different aspects of tactile information (accuracy, tactile resolution, noise) influence learning and performance in the object manipulation tasks. To this end, we have reproduced the experiments without touch sensors (Plappert et al., 2018) and conducted new experiments with touch-sensor readings.

### 2.5 Experimental Analysis Techniques

**FIGURE A1:**
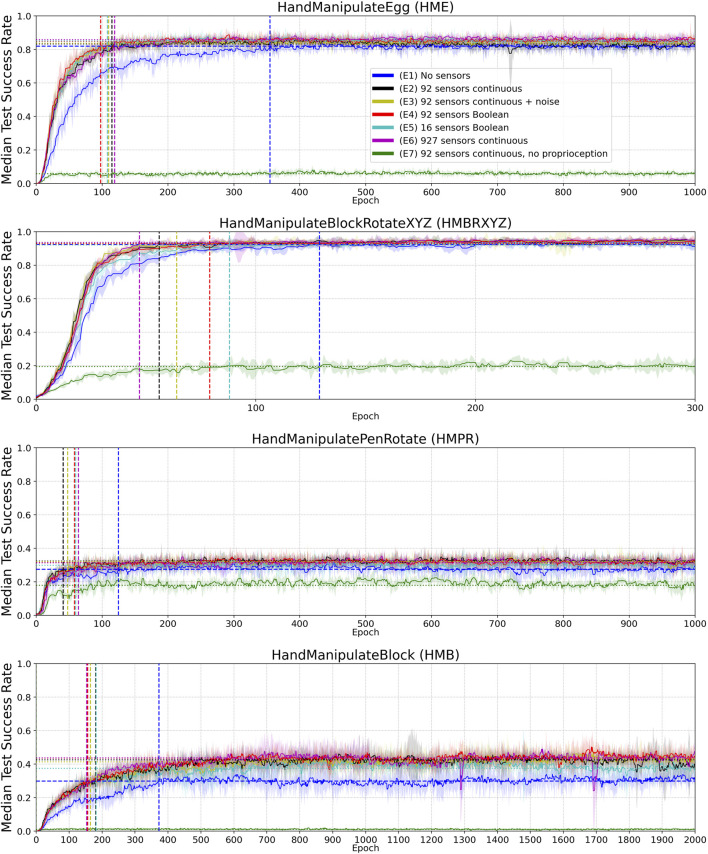
Full version of Figure 4.

To measure performance in the experiments, we calculated a histogram of success-rate values of the median curve of five seeds (per experiment and per environment). You can imagine it as all points of the curves are projected onto the Y axis in Appendix Figure A1 to calculate the histograms. For HME, HMBRXYZ, HMPR, and HMB tasks we used 1,000, 300, 1,000, and 2,000 success-rate values (epochs) respectively to calculate the histograms (Figure 4 and Figure A1). Each histogram has 50 bins between min and max success-rate values. The bin with the maximal number of success-rate values (epochs) was selected as the convergence success-rate level (horizontal dotted and dashed lines in Figure 4). These values are shown in Table 4.

**TABLE 4:** Experiments E1–E7 (Figure 4): Success Rate Convergence Levels (columns 2–8) and Performance Ratio (columns 9–15).

	(E1) No sensors	(E2) 92 sensors continuous	(E3) 92 sensors continuous + noise	(E4) 92 sensors Boolean	(E5) 16 sensors Boolean	(E6) 927 sensors continuous	(E7) 92 sensors continuous + no proprio	(E1) No sensors	(E2) 92 sensors continuous	(E3) 92 sensors continuous + noise	(E4) 92 sensors Boolean	(E5) 16 sensors Boolean	(E6) 927 sensors continuous	(E7) 92 sensors continuous + no proprio
HandManipulate Egg	0.82	0.83	0.84	0.85	0.85	0.86	0.05	1.0	1.01	1.02	1.03	1.04	1.05	0.06
HandManipulate BlockRotateXYZ	0.92	0.93	0.93	0.93	0.92	0.94	0.2	1.0	1.01	1.01	1.01	1.0	1.01	0.21
HandManipulate PenRotate	0.27	0.32	0.32	0.31	0.3	0.31	0.18	1.0	1.18	1.17	1.15	1.09	1.15	0.65
HandManipulate Block	0.3	0.43	0.41	0.44	0.38	0.44	0.01	1.0	1.42	1.38	1.46	1.25	1.46	0.04
Mean								1.0	1.16	1.15	1.16	1.09	1.17	0.24

To measure sample efficiency, we defined *convergence epochs* at which curves (after smoothing to avoid early single-epoch spikes) first intersect with the convergence *success-rate level* of the E1 experiment without tactile sensors (blue horizontal dashed lines in Figure 4). For the smoothing, we used the median curves of the five seeds with a median smoothing window of five epochs (the smoothed curves are plotted in Figure 4). The resulting *convergence epochs* are shown by vertical dashed lines in Figure 4 and represented as numerical values in Table 4.

## 3 Results

In the first experiment (E1), we reproduced the original experiment without touch sensors (“No sensors” in Figure 4) from Plappert et al. (2018). This experiment can be reproduced using the following OpenAI Gym robotics environments:

• “HandManipulateEgg-v0”
• “HandManipulatePenRotate-v0”
• “HandManipulateBlock-v0”
• “HandManipulateBlockRotateXYZ-v0”

In the second experiment (E2), we added continuous-valued sensor readings from 92 sensors to the state (“92 sensors continuous” in Figure 4). The continuous variables have the magnitude of the force detected by the tactile sensors. This experiment can be reproduced using the new OpenAI Gym robotics environments:

• “HandManipulateEggTouchSensors-v1”
• “HandManipulatePenRotateTouchSensors-v1”
• “HandManipulateBlockTouchSensors-v1”
• “HandManipulateBlockRotateXYZTouchSensors-v1”

In the third experiment (E3), we added noise and distortion to the continuous-valued sensor readings using a model with three sources of noise and distortion (“92 sensors continuous + noise” in Figure 4). The first source is a deterministic smoothing of influence of neighboring sensors. Each active sensor distributes 20% of its continuous-valued sensor reading to neighboring sensors in equal proportions. For example, if a tactile sensor has four neighbors, then each of the neighbors accumulates 5% of the continuous-value of the tactile sensor, and the tactile sensor itself gets a reduction of its continuous-value by 20%. The second source is a random noise (standard normal distribution) with standard deviation equal to 1% of the median amplitude of non-zero continuous-value sensor readings. The third source of distortion is the natural logarithm log(V+1) function, where V is a vector of touch values after the deterministic smoothing and random noise distortions. The V values are always greater or equal to zero.

In the fourth experiment (E4), we added Boolean-value sensor readings from 92 sensors to the state (“92 sensors Boolean” in Figure 4). If the tactile sensor detected the force value greater than zero, then for this sensor the environment returns 1, otherwise 0. This experiment can be reproduced using the new OpenAI Gym robotics environments:

• “HandManipulateEggTouchSensors-v0”
• “HandManipulatePenRotateTouchSensors-v0”
• “HandManipulateBlockTouchSensors-v0”
• “HandManipulateBlockRotateXYZTouchSensors-v0”

In the fifth experiment (E5), we grouped 92 sensors into 16 sub-groups (Table 1) to reduce the tactile sensory resolution when using Boolean tactile signals. If any of the sensors in a group has a greater than zero value, then the tactile sub-group returns 1, otherwise 0. The grouping was done per phalanx (3 phalanxes × 5 digits) plus a palm resulting in 16 sub-groups (Table 1). Thus in the fifth experiment, we added Boolean-value readings from the 16 sub-groups to the state (“16 sensors Boolean” in Figure 4).

In the sixth experiment (E6), we added continuous-valued sensor readings from 927 sensors to the state (“(E6) 927 sensors continuous” in Figure 4). The continuous variables have the magnitude of the force detected by the tactile sensors.

In the seventh experiment (E7), we excluded joint angles (24 values, Table 1) and joint angles’ velocity (24 values, Table 1) information from the state and added continuous-valued sensor readings from 92 sensors to the state (“92 sensors continuous, no proprioception” in Figure 4). The continuous variables have the magnitude of the force detected by the tactile sensors.

**FIGURE 5:**
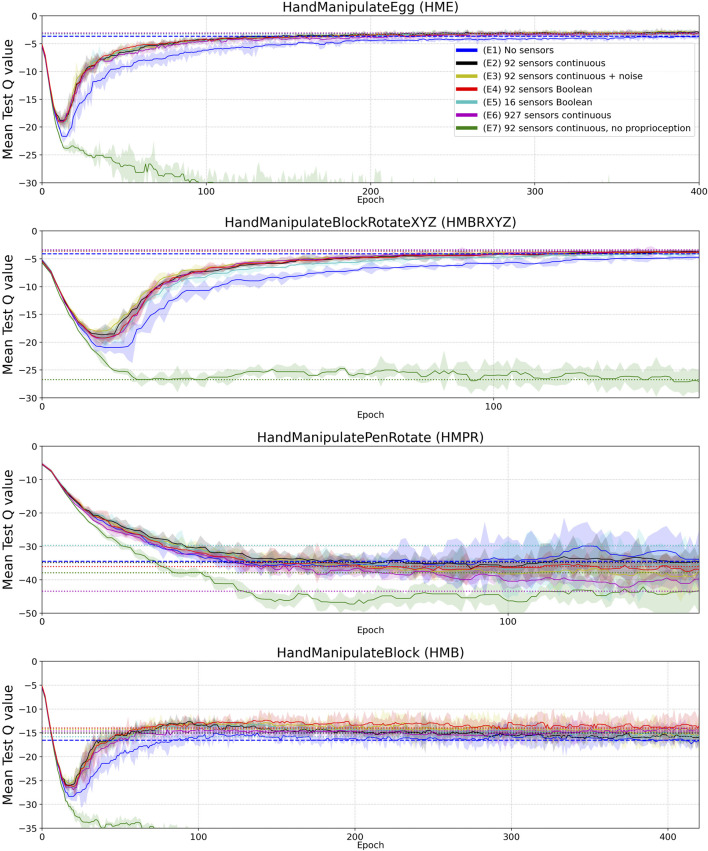
Mean Q value curves (vertical axes) vs. training epochs (horizontal axes) for tasks HME, HMBRXYZ, HMPR, HMB and experiments E1-E7 (colored curves–see HME figure legend). See the full version of this figure in Appendix Figure A2. Each curve shows the median Q-value across five different training trials with a different random seed. The Q-value in each training trial is the mean of 10 tests after each training epoch. The median smoothing window of plotted curves is equal to five epochs. Shaded areas represent the interquartile range. Horizontal dotted lines highlight the mean-Q-value convergence level of corresponding (color) curves.

Figures 4, 5 and results in Table 4 demonstrate that tactile information increases the performance of the agent in the tasks. Results in Table 5 demonstrate sample-efficiency increase while training when tactile information is available. To compare the sample efficiency for learning with and without tactile information, we measured how many training epochs were necessary to reach the performance level of an agent trained without tactile information in an environment. The results indicate more than 2 times faster convergence when tactile information is available (Table 5).

**TABLE 5:** Experiments E1–E7 (Figure 4): Convergence Epoch (columns 2–8) and Sample-Efficiency Ratio (columns 9–15).

	(E1) No sensors	(E2) 92 sensors continuous	(E3) 92 sensors continuous + noise	(E4) 92 sensors Boolean	(E5) 16 sensors Boolean	(E6) 927 sensors continuous	(E7) 92 sensors continuous + no proprio	(E1) No sensors	(E2) 92 sensors continuous	(E3) 92 sensors continuous + noise	(E4) 92 sensors Boolean	(E5) 16 sensors Boolean	(E6) 927 sensors continuous	(E7) 92 sensors continuous + no proprio
HandManipulate Egg	355	115	110	98	108	115	nan	1.0	3.09	3.23	3.62	3.29	3.09	nan
HandManipulate BlockRotateXYZ	129	56	64	79	88	47	nan	1.0	2.3	2.02	1.63	1.47	2.75	nan
HandManipulate PenRotate	125	41	48	58	60	51	nan	1.0	3.05	2.6	2.15	2.08	2.45	nan
HandManipulate Block	373	181	165	156	182	153	nan	1.0	2.06	2.26	2.39	2.05	2.44	nan
Mean								1.0	2.62	2.53	2.45	2.22	2.68	nan

**FIGURE 6:**
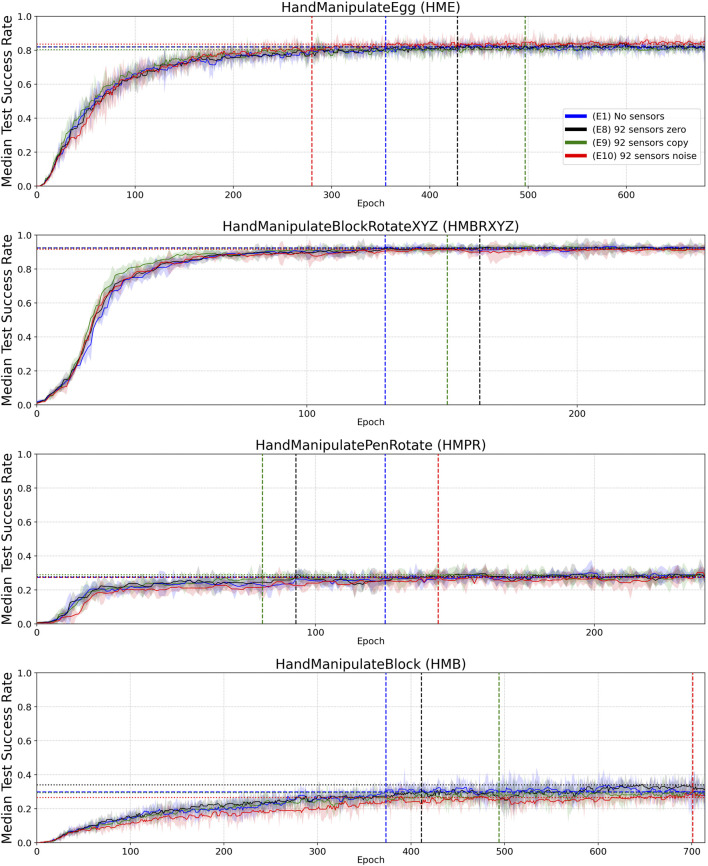
Similar to Figure 4, but for Experiments E8 (black—92 sensors zero), E9 (green—92 sensors copied) and E10 (red—92 sensors noise), with Experiment E1 (blue–no sensors) included again for reference. Median Success Rate curves (vertical axes) vs. training epochs (horizontal axes). Each curve shows the median success rate across five different training trials with a different random seed. The success rate value in each training trial is the mean of 10 tests after each training epoch. The median smoothing window of plotted curves is equal to five epochs. Shaded areas represent the interquartile range. Horizontal dotted lines highlight the success-rate-convergence level of corresponding (color) curves (Table 6). The blue horizontal dashed line highlights the success-rate-convergence level of learning without touch sensors (No sensors) (Table 7). Vertical dashed lines (blue, black, green, and red) highlight the intersection of corresponding curves (color) with the blue dashed line (Table 7).

When introducing tactile data (92 touch sensors), the dimensionality of the state increases from 68 to 160. This increases the number of parameters in the first layer of the neural network (92 (tactile sensors) × 256 (first hidden layer) × 2 (actor, critic) = 47,104). To address a possible hypothesis that the improvement from tactile data could be partially or completely related to the extra parameters in the neural network, we conducted the following three experiments E8-E10 (Tables 6 and 7, Figure 6), which did not confirm this hypothesis:

• E8: ”92 sensors zero” all sensor values are substituted with zeros.
• E9: ”92 sensors copy” all sensor values are substituted by several copies of Joint angles and Joint angles’ velocity (Table 1).
• E10: ”92 sensors noise” all sensor values are substituted by random noise (numpy.random.rand(92))

**TABLE 6:** Experiments E8–E10 (Figure 6): Success Rate Convergence Levels (columns 2–5) and Success Rate Convergence Level Ratio (columns 6–9).

	(E1) No sensors	(E8) 92 sensors zero	(E9) 92 sensors copy	(E10) 92 sensors noise	(E1) No sensors	(E8) 92 sensors zero	(E9) 92 sensors copy	(E10) 92 sensors noise
HandManipulate Egg	0.82	0.82	0.80	0.84	1.0	1.00	0.98	1.02
HandManipulate BlockRotateXYZ	0.92	0.92	0.92	0.92	1.0	1.00	0.99	0.99
HandManipulate PenRotate	0.27	0.28	0.29	0.27	1.0	1.01	1.06	1.00
HandManipulate Block	0.30	0.34	0.29	0.27	1.0	1.14	0.98	0.89
Mean					1.0	**1.04**	1.00	0.97

**TABLE 7:** Experiments E8–E10 (Figure 6): Convergence Epoch (columns 2–5) and Convergence-Epoch Ratio (columns 6–9).

	(E1) No sensors	(E8) 92 sensors zero	(E9) 92 sensors copy	(E10) 92 sensors noise	(E1) No sensors	(E8) 92 sensors zero	(E9) 92 sensors copy	(E10) 92 sensors noise
HandManipulate Egg	355	428	497	280	1.0	0.83	0.71	1.27
HandManipulate BlockRotateXYZ	129	164	152	294	1.0	0.79	0.85	0.44
HandManipulate PenRotate	125	93	81	144	1.0	1.34	1.54	0.87
HandManipulate Block	373	411	494	701	1.0	0.91	0.76	0.53
Mean					**1.0**	0.97	0.97	0.78

**FIGURE 7:**
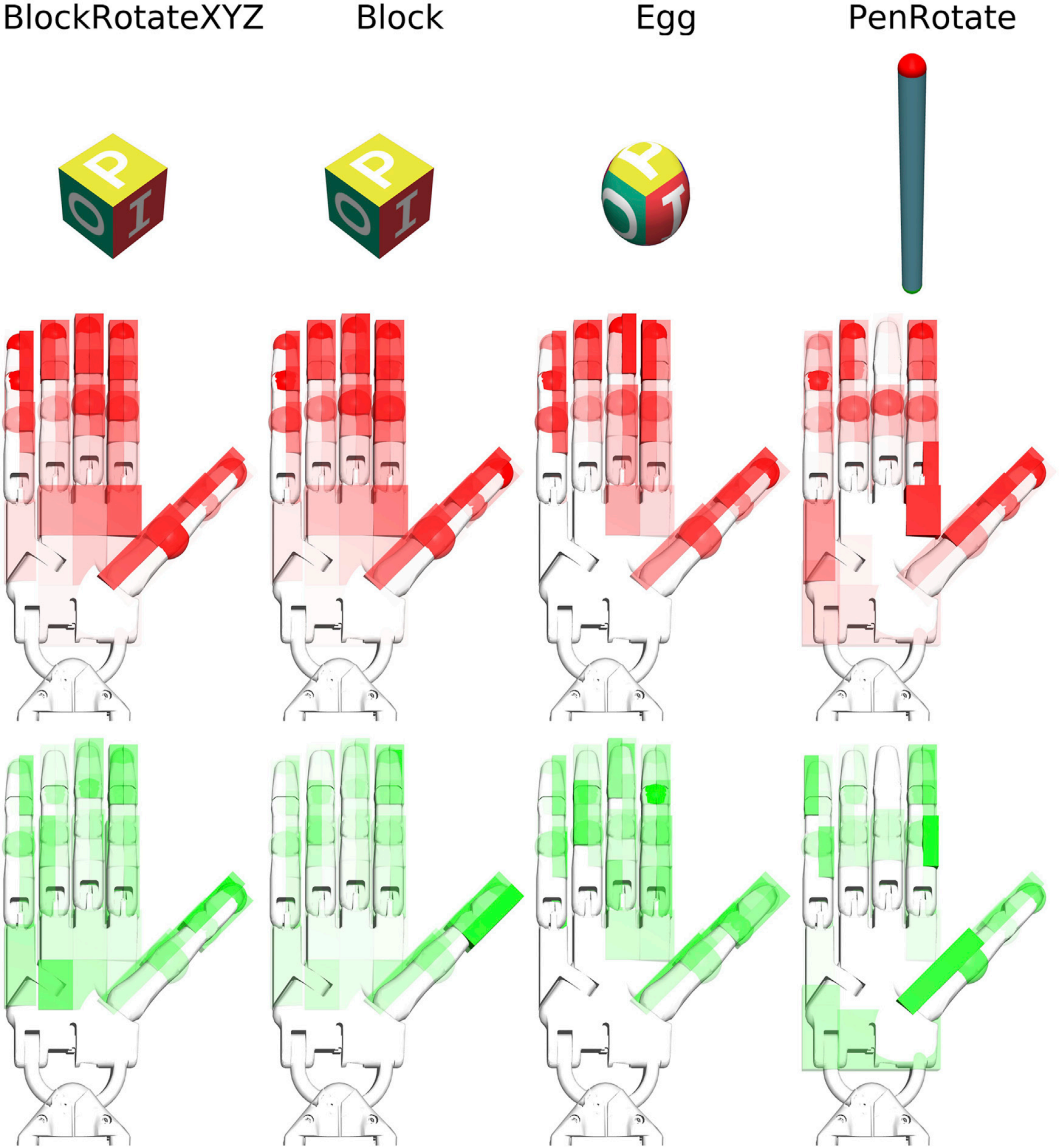
Visualisation of sensors activation (red) and Q-difference (green) values from Table 8. Values are normalized for each hand image.

To obtain some insight about the influence of sensor layouts and sensor density, in the eleventh experiment (E11), we calculated the number of activations per sensor in the 92-sensors layout in the four tasks (Egg-, BlockRotationXYX-, PenRotation-, Block-TouchSensors-v0). To assess the importance of sensory information on different sensors, we also calculated the difference in Q values when dropping out an active sensor (with a Boolean sensor value) (Table 8, Figure 7). We ran 100 episodes per task, with five different trained weights (5 × 20). In the dropping out procedure, we modified the activation value of an active sensor from 1 to 0 and measured the absolute Q-value difference for that modification $\left|Q\left(s_{orig},a_{orig}\right)-Q\left(s_{mod},a_{orig}\right)\right|$. Thus, delta Q arises from the changed input state $s_{mod}$ with the deactivated touch sensor. We set the Q-difference value to nan in Table 8 if a sensor had less than 25 activations in 100 episodes of a task (one activation per four episodes), or if a sensor had less than 50 activations in 400 episodes in all four tasks.

**TABLE 8:** Experiment E11. See the visualization of the values in Figure 7.

Sensor name	Activations all	Activations PenRotate	Activations Egg	Activations Block	Activations BlockRotateXYZ	Mean Q diff all	Mean Q diff PenRotate	Mean Q diff Egg	Mean Q diff Block	Mean Q diff BlockRotateXYZ
Palm 0	80	36	0	22	22	0.12	0.22	nan	nan	nan
Palm 1	99	16	0	50	33	0.15	nan	nan	0.18	0.18
Palm 2	270	0	0	144	126	0.11	nan	nan	0.11	0.11
Palm 3	234	0	0	162	72	0.14	nan	nan	0.18	0.07
Palm 4	489	0	0	266	223	0.09	nan	nan	0.09	0.08
Palm 5	875	0	210	358	307	0.08	nan	0.08	0.08	0.08
Palm 6	1806	538	91	675	502	0.09	0.11	0.08	0.08	0.07
Palm 7	58	56	0	1	1	0.22	0.22	nan	nan	nan
IF PP FLB	689	4	156	323	206	0.1	nan	0.1	0.11	0.06
IF PP FRB	884	453	108	235	88	0.11	0.09	0.13	0.2	0.11
IF PP FLT	1,669	71	503	750	345	0.11	0.12	0.08	0.14	0.07
IF PP FRT	1,021	103	146	525	247	0.19	0.54	0.11	0.18	0.1
IF PP BL	309	83	43	132	51	0.16	0.21	0.05	0.15	0.15
IF PP BR	62	60	0	2	0	0.29	0.28	nan	nan	nan
IF PP Tip	1,235	133	118	703	281	0.12	0.2	0.05	0.11	0.05
IF MP FL	1,517	3	133	845	536	0.11	nan	0.06	0.13	0.07
IF MP FR	809	51	38	519	201	0.13	0.23	0.11	0.14	0.07
IF MP BL	7	5	0	1	1	nan	nan	nan	nan	nan
IF MP BR	27	20	0	4	3	nan	nan	nan	nan	nan
IF MP Tip	570	41	25	395	109	0.1	0.17	0.23	0.1	0.06
IF DP FL	2,267	193	698	929	447	0.16	0.22	0.07	0.22	0.14
IF DP FR	1,604	233	217	778	376	0.19	0.28	0.09	0.25	0.13
IF DP BL	21	4	1	12	4	nan	nan	nan	nan	nan
IF DP BR	113	49	0	49	15	0.33	0.31	nan	0.44	nan
IF DP Tip	2,913	415	657	1,197	644	0.14	0.18	0.06	0.19	0.12
MF PP FLB	327	4	101	132	90	0.08	nan	0.12	0.07	0.04
MF PP FRB	517	10	207	196	104	0.1	nan	0.06	0.14	0.06
MF PP FLT	828	51	103	437	237	0.07	0.16	0.07	0.07	0.04
MF PP FRT	1,609	87	330	789	403	0.11	0.16	0.07	0.14	0.09
MF PP BL	253	83	17	128	25	0.17	0.2	nan	0.14	0.1
MF PP BR	426	75	88	216	47	0.14	0.11	0.11	0.18	0.16
MF PP Tip	1,501	177	190	816	318	0.05	0.06	0.05	0.06	0.03
MF MP FL	552	0	3	289	260	0.07	nan	nan	0.08	0.05
MF MP FR	1,109	0	93	693	323	0.1	nan	0.08	0.12	0.06
MF MP BL	0	0	0	0	0	nan	nan	nan	nan	nan
MF MP BR	68	0	0	68	0	0.17	nan	nan	0.17	nan
MF MP Tip	324	0	9	279	36	0.1	nan	nan	0.1	0.12
MF DP FL	1,280	3	115	676	486	0.11	nan	0.12	0.12	0.09
MF DP FR	2,654	17	883	1,201	553	0.12	nan	0.07	0.17	0.12
MF DP BL	167	3	14	86	64	0.08	nan	nan	0.09	0.06
MF DP BR	234	2	25	202	5	0.18	nan	0.12	0.18	nan
MF DP Tip	3,377	12	760	1,694	911	0.08	nan	0.05	0.11	0.07
RF PP FLB	146	0	0	81	65	0.12	nan	nan	0.12	0.12
RF PP FRB	262	7	13	137	105	0.06	nan	nan	0.05	0.04
RF PP FLT	154	48	13	62	31	0.18	0.14	nan	0.19	0.14
RF PP FRT	613	92	88	299	134	0.1	0.15	0.06	0.1	0.07
RF PP BL	22	18	0	4	0	nan	nan	nan	nan	nan
RF PP BR	355	62	78	187	28	0.12	0.2	0.08	0.12	0.05
RF PP Tip	705	150	107	367	81	0.09	0.1	0.08	0.09	0.06
RF MP FL	191	62	47	61	21	0.15	0.13	0.15	0.22	nan
RF MP FR	522	15	115	245	147	0.13	nan	0.08	0.1	0.06
RF MP BL	41	37	0	4	0	nan	0.25	nan	nan	nan
RF MP BR	45	2	7	35	1	nan	nan	nan	0.14	nan
RF MP Tip	311	82	63	108	58	0.12	0.15	0.08	0.14	0.08
RF DP FL	2046	297	301	892	556	0.06	0.05	0.06	0.07	0.05
RF DP FR	1,586	66	515	685	320	0.06	0.12	0.05	0.07	0.05
RF DP BL	124	67	3	30	24	0.06	0.06	nan	0.06	nan
RF DP BR	156	8	10	137	1	0.24	nan	nan	0.22	nan
RF DP Tip	2,988	364	639	1,307	678	0.03	0.05	0.03	0.04	0.02
LF M F	331	171	0	90	70	0.09	0.07	nan	0.14	0.12
LF PP FLB	8	3	0	3	2	nan	nan	nan	nan	nan
LF PP FRB	251	14	1	141	95	0.12	nan	nan	0.14	0.09
LF PP FLT	3	0	0	2	1	nan	nan	nan	nan	nan
LF PP FRT	1,326	64	407	542	313	0.13	0.29	0.1	0.16	0.09
LF PP BL	21	7	0	7	7	nan	nan	nan	nan	nan
LF PP BR	486	65	98	254	69	0.22	0.2	0.2	0.25	0.15
LF PP Tip	975	42	319	452	162	0.11	0.16	0.08	0.14	0.1
LF MP FL	43	13	1	17	12	nan	nan	nan	nan	nan
LF MP FR	1,669	73	395	700	501	0.09	0.09	0.07	0.12	0.08
LF MP BL	1,120	96	292	494	238	0.07	0.11	0.06	0.07	0.05
LF MP BR	2,839	345	536	1,325	633	0.07	0.05	0.07	0.09	0.05
LF MP Tip	3,106	321	536	1,482	767	0.04	0.05	0.04	0.06	0.03
LF DP FL	60	47	5	5	3	0.31	0.33	nan	nan	nan
LF DP FR	2,188	105	427	906	750	0.12	0.1	0.1	0.14	0.13
LF DP BL	90	16	22	34	18	0.14	nan	nan	0.08	nan
LF DP BR	1,616	63	283	777	493	0.11	0.06	0.08	0.15	0.1
LF DP Tip	1918	79	188	918	733	0.08	0.11	0.05	0.1	0.08
TH PP FL	1,670	68	273	759	570	0.14	0.53	0.08	0.18	0.09
TH PP FR	33	8	0	13	12	nan	nan	nan	nan	nan
TH PP BL	430	4	36	274	116	0.2	nan	0.17	0.25	0.12
TH PP BR	0	0	0	0	0	nan	nan	nan	nan	nan
TH PP Tip	1,699	74	297	785	543	0.13	0.33	0.08	0.15	0.09
TH MP FL	2017	375	349	831	462	0.14	0.19	0.09	0.15	0.09
TH MP FR	17	5	2	8	2	nan	nan	nan	nan	nan
TH MP BL	621	68	35	378	140	0.2	0.12	0.16	0.23	0.15
TH MP BR	8	0	0	8	0	nan	nan	nan	nan	nan
TH MP Tip	982	163	169	483	167	0.12	0.12	0.11	0.15	0.08
TH DP FL	2,273	313	496	1,035	429	0.19	0.21	0.1	0.28	0.15
TH DP FR	169	18	80	62	9	0.23	nan	0.12	0.53	nan
TH DP BL	1,668	401	120	896	251	0.29	0.2	0.12	0.36	0.26
TH DP BR	223	2	17	162	42	0.3	nan	nan	0.35	0.13
TH DP Tip	2,787	329	575	1,467	416	0.19	0.3	0.08	0.25	0.17

Sensors activation (col. 2–6) and Q-difference value (col. 7–11).

IF - Index Finger, MF - Middle Finger, RF - Ring Finger, LF - Little Finger, DP - Distal Phalanx, MP - Middle Phalanx, PP - Proximal Phalanx, FL - Front Left, FLB - Front Left Bottom, FLT - Front Left Top, FR - Front Right, FRB - Front Right Bottom, FRT - Front Right Top, BL - Back Left, BR - Back Right.

## 4 Discussion

In this work, we examined the inclusion of tactile information in model-free deep reinforcement learning and found that tactile information improves sample efficiency of learning and the performance. We take this as corroborating believe that tactile sensing provides information about geometry and contact physics of manipulated objects that is valuable for learning. The exact geometry of the manipulated objects and actuators was not explicitly represented in the state vector of the hand model without tactile sensors. Information about object state was represented via 13 continuous values (Table 1), information about the actuators via 48 continuous values. Touch information may facilitate learning a latent representation that connects this state information with information about object geometry and physics gathered from sensed contacts. Such representation should be useful in in-hand manipulation tasks. Lower tactile-sensor resolution corresponds to lower accuracy of inference for the object’s geometry and contact physics. This assumption fits the observed negative effect of lower tactile-sensors resolution on DRL performance (Table 5).

Boolean-valued sensor readings appear to lead to a final performance and sample efficiency of learning similar to that obtained using continuous sensor values. This may indicate that contact information is most informative in the experiments and force value adds relatively little information to aid the learning process. Likewise, added noise and distortion to sensory readings do not seem to affect learning and performance, although the noise and distortion were moderate, as we set the noise and distortion parameters close to real-world implementations (Figure 2). As we demonstrated in ablation experiments (E8–E10), 100% noise in the tactile signal makes performance similar to the experiments without tactile information and learning less sample efficient (Tables 6 and 7, Figure 6).

The inclusion of additional tactile data increases the dimensionality of the state from 68 to 160. This leads to an increase in the number of parameters in the neural network by 16% or 47,104 parameters (92 (tactile sensors) × 256 (first hidden layer) × 2 (actor, critic)). This marginally increases computation time for DRL training, however, the sample efficiency of learning increases by more than twofold (Table 5).

The reported performance gains with tactile sensors are dependent on the *success-rate level* without tactile sensors. The better the task can be solved without tactile information, the less is the impact of tactile information on the performance gains (Table 4). For example, in the “HandManipulateBlock” environment the success-rate convergence level without tactile information is equal to 0.3 (E1), and equal to 0.43 (E2) with 92 continuous-value sensors, which gives an about 42% performance gain. In the “HandManipulateBlockRotateXYZ” environment the success-rate convergence level without tactile information is equal to 0.92 (E1), and equal to 0.93 (E2) with 92 continuous-value sensors, which gives an about 1% performance gain. Thus, our results show that when an object-manipulation task can be solved without tactile information, tactile information can only add marginal performance gain. However, when information about the position, orientation, velocities of the object is not enough for a confident solution of the task, tactile information can provide a significant improvement to the performance.

Moving from the dimension of performance to sample efficiency, we observe that even in cases where tactile information is unable to significantly contribute to performance (since the task can be solved well in the absence of tactile information already), tactile information can still more than double sampling efficiency (see Table 5).

Finding accurate numeric values for convergence levels of DRL curves within a limited number of training epochs and determining the epoch of the first intersection of the convergence curve with the convergence level is not trivial. The issues include local spikes in convergence curves, low-frequency oscillations around a convergence level, and temporal performance drops. A wide smoothing window may help to remove spikes, but it does not help to remove low-frequency oscillations and temporal performance drops. Moreover, it shifts the first intersection with the convergence curve with the convergence level. Also, it does not solve the issue of how to select the numeric value of the convergence level, for example, it can be the highest value of the smoothed convergence curve. However, the value will depend on the smoothing window width parameter, and the curve does not reach this highest point again in the experiment, thus it is arguable whether it is the convergence level. Another approach is statistical metrics to select the convergence level. One approach would be to find the median value of all success-rate values, but in this case, the duration of the initial slope will influence the level. Thus, we decided to use the histogram of all success-rate values of the convergence curve to find the bin with the most number of samples. You can imagine it as all points of the curves are projected onto the Y axis in Appendix Figure A1 to calculate the histograms. We assume that this approach solves the issues of spikes, low-frequency oscillations, and temporal performance drops. However, one of the issues of this approach is the bin width. One the one hand we want to have narrow bins to have a higher precision of the convergence level. On the other hand, too narrow bins will result in a small number of samples per bin leading to a spiky histogram making the identification of the true level less reliable. We decided to use 50 bins, as a good trade-off between the smoothness of the histogram and the narrowness of the bin size. The so-defined numeric convergence levels are in good visual accordance with the shape of the plotted convergence curves.

The experiment E11 tests the effect of replacing a positive scalar value of an active sensor in a state-action pair, with a scalar value of zero for that sensor (averaged over a lot of state-action pairs and tactile sensors). The results of experiment E11 (Table 8, Figure 7) show that the most frequently activated sensors are not always the most informative in terms of the Q value. However, the thumb and index finger provide the most informative sensory information for in-hand manipulation of objects type of tasks. Of course, this information is only relevant to the degree that the learnt Q-values do indeed contribute to successful trajectories. While the experiment cannot guarantee this for each single case, our employed averaging over a set of tasks that contained a significant proportion of accurately trained trajectories makes us expect that the observed correlations are meaningful.

In a study of a learning dexterous in-hand manipulation (OpenAI et al., 2020) authors used reinforcement learning to learn dexterous in-hand manipulation policies and transferred them to a physical robot despite being trained entirely in simulation. The training was conducted in a simulated MuJoCo environment. In that work, the authors avoided providing the fingertip tactile sensor measures as observations to the policy because that would have been difficult to model provided tactile sensors in the simulator. Indeed, accurate modeling of tactile sensors in a simulator can be a challenging problem. However, simplifications or embedding of tactile information may allow a transfer between real and simulated tactile information. Specifically, our results in experiments E3–E4 demonstrate that noisy or Boolean tactile information provides significant improvements in sample-efficiency of training and performance.

In the HandManipulatePenRotate task, a relatively low median test success rate was obtained, compared to other tasks. The reason for this is that, depending on the combination of initial and goal pen orientation, we get different degrees of difficulty, where in the simplest case the task leads to a moderate adjustment of the pen angle, and in the most difficult case the pen needs to be rotated 180°. In this more difficult case, the agent failed to learn the necessary manipulation. Consequently, the agent was unable to solve about half of the initial conditions, but was able to fulfill most cases with moderate pen orientation adjustments. In the HandManipulateBlock task, we also got a relatively low median test success rate compared to HandManipulateBlockRotateXYZ. The reason for this is that, depending on the combination of the initial and goal positions of the block, we get a different degree of manipulation complexity, while in the HandManipulateBlockRotateXYZ task, the agent was able to choose the most reliable position for the manipulated block, given a fixed wrist.

Computational resources are the main limiting factor of this study. The shown convergence curves are approximately equal to one year of computing time for one machine with 20 CPU cores (see Section 2.4). With more processing power, more experiments could be done to reveal the effect of tactile information on DRLs in dynamic in-hand manipulation tasks. Interesting directions for future work include: hierarchical (Schilling and Melnik, 2018), causal (Melnik et al., 2019a), and modular (Konen et al., 2019; Melnik et al., 2019b) tactile representations; sensor ablation studies to shed light on the role of sensor placement; the impact of different types of noise; improvements with tactile sensors using various DRL algorithms (Bach et al., 2020; Kidzinski et al., 2018) such as soft actor-critic (SAC) (Haarnoja et al., 2018) or twin delayed deep deterministic (TD3) (Fujimoto et al., 2018). It would also be interesting to draw connections to tactile representations in terms of embodied cognition (Peter et al., 2018) and theories of sensorimotor processing (Melnik et al., 2017; Melnik, 2017) in the human brain.

## 5 Conclusion

The paper investigates the learning performance of DRL algorithms with and without touch information for the dexterous in-hand object manipulation tasks. The DRL algorithms investigated in the work are DDPG and HER techniques, and the robot hand used in the work is a simulation of the Shadow Dexterous Hand in the OpenAI Gym robotics environment, which we extended by an arrangement of touch sensors (Koiva et al., 2013; Büscher et al., 2015). We concatenated all experiments using the MuJoCo physics engine (Todorov et al., 2012). It was found that the touch information can increase sample efficiency and performance of the DRL algorithms.

In this work, we introduce the touch-sensors extensions to OpenAI Gym (Brockman et al., 2016) () robotics Shadow-Dexterous-Hand environments (Plappert et al., 2018) modeled after our touch sensor developments (Koiva et al., 2013; Büscher et al., 2015). We concatenated tactile, proprioceptive, and visual information at the input level of a neural network. We find that adding normal-force tactile sensing data can produce a more than threefold increase in sample efficiency (Table 5) and performance gains of up to 46% (Table 4: E4-, E6-HandManipulateBlock) when training with DDPG + HER on several simulated manipulation tasks. To examine the role of tactile-sensor parameters in these improvements, we conducted experiments (Figure 4) with varied sensor-measurement accuracy (ground truth continuous values, noisy continuous values, Boolean values), and varied spatial resolution of the tactile sensors (92 sensors vs. 16 pooled sensor areas on the hand). We conclude that the observed benefits of tactile input on sample efficiency are rather robust against changes in resolution, signal binarization, distortion, or (limited) noise. As a remarkable result, we found binary contact detection on par with providing accurate continuous contact-normal-force values when training with deep reinforcement learning techniques. However, dense tactile resolution may help to improve performance and sample efficiency. These findings may provide guidance for those looking to build robots with tactile sensors for object manipulation.

A possible further extension of this work is multi-modal sensor fusion. The multi-modal sensor fusion (Korthals et al., 2019b) allows end-to-end training of Bayesian information fusion on raw data for all subsets of a sensor setup. It can potentially deliver better performance and more sample efficient training with model-free deep reinforcement learning approaches.

## Figures and Tables

**FIGURE A2:**
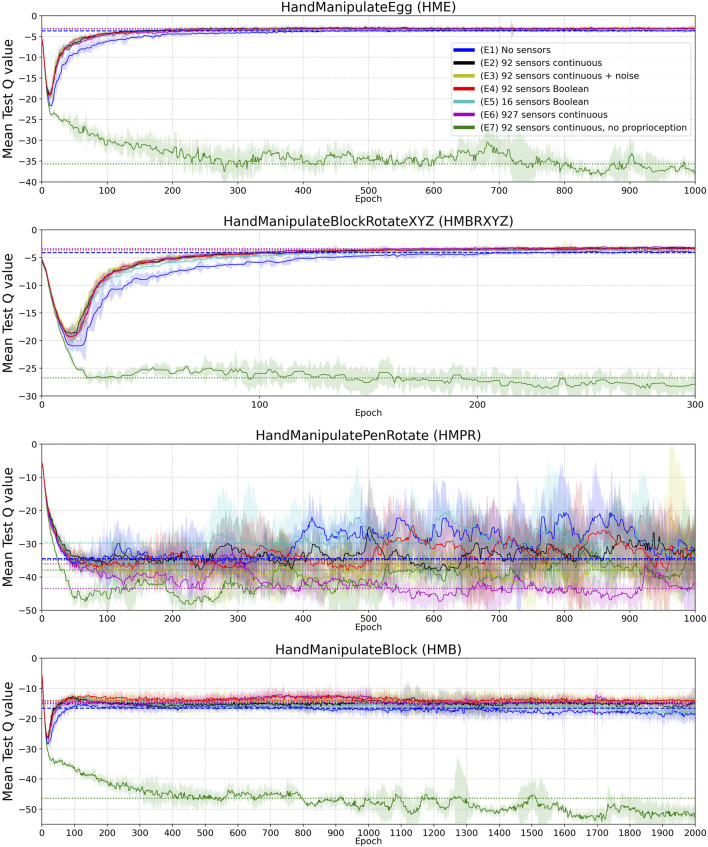
Full version of Figure 5.

## Data Availability

The datasets generated for this study are available on request to the corresponding author. The experiments can be reproduced using OpenAI Gym environments (https://github.com/openai/gym) listed in Table 3. Video: https://rebrand.ly/TouchSensors

## Footnotes

1 OpenAI Baselines. URL: https://github.com/openai/baselines.

2 OpenAI Gym. URL: https://github.com/openai/gym.

## References

[B1] Andrychowicz M. Wolski F. Ray A. Schneider J. Fong R. Welinder P. (2017). “Hindsight Experience Replay,” in Advances in Neural Information Processing Systems, 5048–5058.

[B2] Bach N. Melnik A. Schilling M. Korthals T. Ritter H. (2020). “Learn to Move through a Combination of Policy Gradient Algorithms: Ddpg, D4pg, and Td3,” in International Conference on Machine Learning, Optimization, and Data Science (Berlin: Springer), 631–644. 10.1007/978-3-030-64580-9_52

[B3] Brockman G. Cheung V. Pettersson L. Schneider J. Schulman J. Tang J. (2016). Openai Gym. arXiv. preprint: arXiv:1606.01540.

[B4] Büscher G. H. Kõiva R. Schürmann C. Haschke R. Ritter H. J. (2015). Flexible and Stretchable Fabric-Based Tactile Sensor. Rob. Autonomous Syst. 63, 244–252. 10.1016/j.robot.2014.09.007

[B5] Calandra R. Owens A. Jayaraman D. Lin J. Yuan W. Malik J. (2018). More Than a Feeling: Learning to Grasp and Regrasp Using Vision and Touch. IEEE Robot. Autom. Lett. 3 (4), 3300–3307. 10.1109/lra.2018.2852779

[B6] Chebotar Y. Kroemer O. Peters J. (2014). “Learning Robsot Tactile Sensing for Object Manipulation,” in 2014 IEEE/RSJ International Conference on Intelligent Robots and Systems, Chicago, IL,, September 14-18, 2014 (IEEE), 3368–3375.

[B7] Church A. Lloyd J. Hadsell R. Lepora N. F. (2020). Deep Reinforcement Learning for Tactile Robotics: Learning to Type on a Braille Keyboard. IEEE Robot. Autom. Lett. 5, 6145–6152. 10.1109/lra.2020.3010461

[B8] Dang H. Weisz J. Allen P. K. (2011). “Blind Grasping: Stable Robotic Grasping Using Tactile Feedback and Hand Kinematics,” in ICRA, Shanghai, China, May 9-13, 2011, 5917–5922.

[B9] Fritzsche M. Elkmann N. Schulenburg E. (2011). “Tactile Sensing: A Key Technology for Safe Physical Human Robot Interaction,” in 2011 6th ACM/IEEE International Conference on Human-Robot Interaction (HRI), Lausanne, Switzerland, March 8-11, 2011 (IEEE), 139–140.

[B10] Fujimoto S. van Hoof H Meger D. (2018). Addressing Function Approximation Error in Actor-Critic Methods. Proc. Machine Learn. Res. 80. 1587–1596.

[B11] Gary S. (2008a). “Xenocollide: Complex Collision Made Simple,” in Game programming Gems 7 (Course Technology), 165–178.

[B12] Gary S. (2008). Minkowski Portal Refinement in 2D.

[B13] Haarnoja T. Zhou A. Abbeel P. Levine S. (2018). “Soft Actor-Critic: Off-Policy Maximum Entropy Deep Reinforcement Learning with a Stochastic Actor,” in International Conference on Machine Learning, 1861–1870.

[B14] Johansson R. S. Westling G. (1984). Roles of Glabrous Skin Receptors and Sensorimotor Memory in Automatic Control of Precision Grip when Lifting Rougher or More Slippery Objects. Exp. Brain Res. 56 (3), 550–564. 10.1007/BF00237997 6499981

[B16] Kidzi´nski Ł. Mohanty S. P. Huang Z. Ong C. Zhou S. Pechenko A. (2018). “Learning to Run challenge Solutions: Adapting Reinforcement Learning Methods for Neuromusculoskeletal Environments,” in The NIPS’17 Competition: Building Intelligent Systems (Springer), 121–153.

[B17] Kingma D. P. Jimmy B. (2014). Adam: A Method for Stochastic Optimization. arXiv. preprint: arXiv:1412.6980.

[B18] Koiva R. Zenker M Schurmann C. Haschke R. Ritter H. J. (2013). “A Highly Sensitive 3D-Shaped Tactile Sensor,” in 2013 IEEE/ASME International Conference on Advanced Intelligent Mechatronics (IEEE), Wollongong, NSW, July 9-12, 2013, 1084–1089.

[B19] Konen K. Korthals T. Melnik A. Schilling M. (2019). “Biologically-comment inspired Deep Reinforcement Learning of Modular Control for a Sixlegged Robot,” in 2019 IEEE International Conference on Robotics and Automation Workshop on Learning Legged Locomotion Workshop, (ICRA) 2019, Montreal, CA, May 20–25, 2019.

[B20] Korthals T. Melnik A. Hesse M. Leitner J. (2019). “Multisensory Assisted In-Hand Manipulation of Objects with a Dexterous Hand,” in 2019 IEEE International Conference on Robotics and Automation Workshop on Integrating Vision and Touch for Multimodal and Cross-modal Perception, (ViTac), Montreal, CA, May 20-25, 2019.

[B21] Korthals T. (2019). “Jointly Trained Variational Autoencoder for Multi-Modal Sensor Fusion,” in 2019 22st International Conference on Information Fusion, (FUSION), Ottawa, CA, July 2–5, 2019.

[B22] Kumar V. Todorov E. Levine S. (2016). “Optimal Control with Learned Local Models: Application to Dexterous Manipulation,” in 2016 IEEE International Conference on Robotics and Automation (ICRA), Stockholm, Sweden, May 16-21, 2016 (IEEE), 378–383.

[B23] Lederman S. J. Klatzky R. L. (1987). Hand Movements: A Window into Haptic Object Recognition. Cogn. Psychol. 19 (3), 342–368. 10.1016/0010-0285(87)90008-9 3608405

[B24] Lillicrap T. P. Hunt J. J. Pritzel A. Heess N. Erez T. Tassa Y. (2016). “Continuous Control with Deep Reinforcement Learning,” in ICLR (Poster).

[B25] Maycock J. Dornbusch D. Elbrechter C. Haschke R. Schack T. Ritter H. (2010). Approaching Manual Intelligence. Künstl Intell. 24 (4), 287–294. 10.1007/s13218-010-0064-9

[B26] Meier M. Walck G. Haschke R. Ritter H. J. (2016). “Distinguishing Sliding from Slipping during Object Pushing,” in 2016 IEEE/RSJ International Conference on Intelligent Robots and Systems (IROS), Daejeon, South Korea, October 9-14, 2016 (IEEE), 5579–5584.

[B27] Melnik A. Hairston W. D. Ferris D. P. König P. (2017). EEG Correlates of Sensorimotor Processing: Independent Components Involved in Sensory and Motor Processing. Sci. Rep. 7 (1), 4461–4515. 10.1038/s41598-017-04757-8 28667328 PMC5493645

[B28] Melnik A. Bramlage L. Voss H. Rossetto F. Ritter H. (2019). “Combining Causal Modelling and Deep Reinforcement Learning for Autonomous Agents in Minecraft,” in 4th Workshop on Semantic Policy and Action Representations for Autonomous Robots at IROS 2019, Macau, China, November 8, 2019.

[B29] Melnik A. Fleer S. Schilling M. Ritter H. (2019). Modularization of End-To-End Learning: Case Study in arcade Games. arXiv. preprint: arXiv:1901.09895.

[B30] Melnik A. (2017). Sensorimotor Processing in the Human Brain and Cognitive Architecture. PhD thesis Universität Osnabrück.

[B31] Merzić H. Bogdanovic M. Kappler D. Righetti L. Bohg J. (2019). “Leveraging Contact Forces for Learning to Grasp,” in International Conference on Robotics and Automation (ICRA) Montreal, QC, May 20-24, 2019 (IEEE), 3615–3621.

[B15] Michael J. Newth O. (2013). “Minkowski Portal Refinement and Speculative Contacts in Box2D. San Jose, CA: San Jose State University.

[B32] OpenAI Andrychowicz O. M. Baker B. Chociej M. Józefowicz R. McGrew B. Pachocki J. (2020). Learning Dexterous In-Hand Manipulation. Int. J. Rob. Res. 39 (1), 3–20. 10.1177/0278364919887447

[B33] Peter K. Melnik A. Goeke C. Gert A. L. König S. U. Kietzmann T. C. (2018). “Embodied Cognition,” in 2018 6th International Conference on Brain-Computer Interface (BCI) (IEEE), 1–4.

[B34] Plappert M. Andrychowicz M. Ray A. McGrew B. Baker B. Powell G. (2018). Multi-goal Reinforcement Learning: Challenging Robotics Environments and Request for Research. arXiv. preprint: arXiv:1802.09464.

[B35] Rajeswaran A. Kumar V. Gupta A. Vezzani G. Schulman J. Todorov E. (2017). Learning Complex Dexterous Manipulation with Deep Reinforcement Learning and Demonstrations. arXiv. preprint arXiv:1709.10087.

[B36] Schilling M. Melnik A. (2018). “An Approach to Hierarchical Deep Reinforcement Learning for a Decentralized Walking Control Architecture,” in Biologically Inspired Cognitive Architectures Meeting (Berlin: Springer), 272–282. 10.1007/978-3-319-99316-4_36

[B37] Schmitz A. Bansho Y. Noda K. Iwata H. Ogata T. Sugano S. (2014). “Tactile Object Recognition Using Deep Learning and Dropout,” in 2014 IEEE-RAS International Conference on Humanoid Robots, Madrid, Spain, November 18-20, 2014 (IEEE), 1044–1050.

[B38] Tian S. Ebert F. Jayaraman D. Mudigonda M. Finn C. Calandra R. (2019). “Manipulation by Feel: Touchbased Control with Deep Predictive Models,” in 2019 International Conference on Robotics and Automation (ICRA), Montreal, QC, May 20-24, 2019 (IEEE), 818–824.

[B39] Todorov E. (2019). Sensor/Touch. URL: http://www.mujoco.org/book/XMLreference.html#sensor-touch.

[B40] Todorov E. (2019). MPL Sensors. URL: http://www.mujoco.org/book/haptix.html#mpSensors.

[B41] Todorov E. Tom E. Tassa Y. (2012). “Mujoco: A Physics Engine for Model-Based Control,” in 2012 IEEE/RSJ International Conference on Intelligent Robots and Systems, Vilamoura-Algarve, Portugal, October 7-12 , 2012 (IEEE), 5026–5033.

[B42] Tom S. Horgan D. Gregor K. Silver D. (2015). “Universal Value Function Approximators,” in International conference on machine learning, 1312–1320.

[B43] Van Hoof H. Hermans T. Neumann G. Peters J. (2015). “Learning Robot In-Hand Manipulation with Tactile Features,” in 2015 IEEE-RAS 15th International Conference on Humanoid Robots (Humanoids), Seoul, South Korea, November 3-5, 2015 (IEEE), 121–127.

[B44] Van Hoof H. Chen N. Karl M. van der Smagt P. Peters J. (2016). “Stable Reinforcement Learning with Autoencoders for Tactile and Visual Data,” in 2016 IEEE/RSJ International Conference on Intelligent Robots and Systems (IROS), Daejeon, South Korea, October 9-14, 2016 (IEEE), 3928–3934.

[B45] Walck G. Haschke R. Meier M. Ritter H. J. (2017). “Robot Self-protection by Virtual Actuator Fatigue: Application to Tendon-Driven Dexterous Hands during Grasping,” in 2017 IEEE/RSJ International Conference on Intelligent Robots and Systems (IROS), Vancouver, BC, September 24-28, 2017 (IEEE), 2200–2205.

